# Symptomatic Trends and Time to Recovery for Long COVID Patients Infected During the Omicron Phase

**DOI:** 10.3390/jcm14144918

**Published:** 2025-07-11

**Authors:** Hiroshi Akiyama, Yasue Sakurada, Hiroyuki Honda, Yui Matsuda, Yuki Otsuka, Kazuki Tokumasu, Yasuhiro Nakano, Ryosuke Takase, Daisuke Omura, Keigo Ueda, Fumio Otsuka

**Affiliations:** Department of General Medicine, Graduate School of Medicine, Dentistry and Pharmaceutical Sciences, Okayama University, 2-5-1 Shikata-cho, Kitaku, Okayama 700-8558, Japan; pdtm94yn@s.okayama-u.ac.jp (H.A.); pzaf6h9w@s.okayama-u.ac.jp (Y.S.); ppgf1hrd@s.okayama-u.ac.jp (H.H.); phvw0350@okayama-u.ac.jp (Y.M.); otsuka@s.okayama-u.ac.jp (Y.O.); tokumasu@okayama-u.ac.jp (K.T.); me421055@s.okayama-u.ac.jp (Y.N.); p4v05asb@okayama-u.ac.jp (R.T.); pv469wdb@okayama-u.ac.jp (D.O.); p02n620b@okayama-u.ac.jp (K.U.)

**Keywords:** fatigue, headache, insomnia, long COVID, Omicron variants, recovery

## Abstract

**Background:** Since the pathophysiology of long COVID is not yet fully understood, there are no specific methods for its treatment; however, its individual symptoms can currently be treated. Long COVID is characterized by symptoms that persist at least 2 to 3 months after contracting COVID-19, although it is difficult to predict how long such symptoms may persist. **Methods:** In the present study, 774 patients who first visited our outpatient clinic during the Omicron period from February 2022 to October 2024 were divided into two groups: the early recovery (ER) group (370 cases; 47.8%), who recovered in less than 180 days (median 33 days), and the persistent-symptom (PS) group (404 cases; 52.2%), who had symptoms that persisted for more than 180 days (median 437 days). The differences in clinical characteristics between these two groups were evaluated. **Results:** Although the median age of the two groups did not significantly differ (40 and 42 in ER and PS groups, respectively), the ratio of female patients was significantly higher in the PS group than the ER group (59.4% vs. 47.3%). There were no significant differences between the two groups in terms of the period after infection, habits, BMI, severity of COVID-19, and vaccination history. Notably, at the first visit, female patients in the PS group had a significantly higher rate of complaints of fatigue, insomnia, memory disturbance, and paresthesia, while male patients in the PS group showed significantly higher rates of fatigue and headache complaints. Patients with more than three symptoms at the first visit were predominant in the PS groups in both genders. Notably, one to two symptoms were predominant in the male ER group, while two to three symptoms were mostly reported in the female PS group. Moreover, the patients in the PS group had significantly higher scores for physical and mental fatigue and for depressive symptoms. **Conclusions:** Collectively, these results suggest that long-lasting long COVID is related to the number of symptoms and presents gender-dependent differences.

## 1. Introduction

COVID-19, which was first reported in Wuhan, China, in late 2019, triggered a global pandemic. As of May 2025, the cumulative number of confirmed COVID-19 cases worldwide has reached 777.7 million, with over 7.1 million deaths [https://ourworldindata.org/grapher/cumulative-covid-cases-region (accessed on 3 July 2025)]. COVID-19 has been shown to cause a variety of symptoms not only in the acute phase after SARS-CoV-2 infection, but also over the long-term. This long-term condition—commonly known as post-COVID-19 condition (PCC), post-acute sequelae of COVID-19 (PASC), or long COVID [1,2]—manifests as a wide range of symptoms, including fatigue, dyspnea, and cognitive dysfunction. The World Health Organization (WHO) defines PCC as a condition characterized by a range of symptoms that usually begin within 3 months of the initial symptoms of COVID-19, persist for at least 2 months, and cannot be explained by any other diagnosis [3].

The pathophysiology of long COVID remains complex, involving microthrombus, inflammation, autoimmunity, viral persistence, neurological damage, and vascular dysfunction [4,5]. This complexity suggests the need for multidisciplinary rather than single therapeutic approaches [6]. Long COVID varies not only in terms of symptoms but also in severity, as some people have mild symptoms while others present symptoms so severe that they interfere with their daily life and employment [7]. The prolongation of poor physical and mental conditions not only reduces an individual’s quality of life (QOL), but also poses serious public health challenges such as an increased burden on the health care system and withdrawal from the labor force.

The reported prevalence of long COVID varies significantly, depending on the study design, target population, and timing of assessment. Approximately 10 to 30% of infected individuals experience persistent symptoms lasting beyond one to three months after infection [8,9,10,11]. The related symptoms can persist for weeks, months, or even longer; global estimates for 2022 indicate that approximately 15% of individuals will still have symptoms at 12 months [12]. In a matched cohort study conducted in Berlin, Germany, most recovered COVID-19 patients regained their health within 6.5 months. On the other hand, symptoms such as taste and smell disturbances, as well as neurological and cognitive impairments (e.g., so-called “brain fog”), require a longer recovery period [13,14].

Middle older age, female gender, acute severity, history of hospitalization, and pre-existing comorbidities have been reported as major risk factors for the persistence of long COVID [15,16,17]. Notably, recent studies have suggested a significant clinical overlap between long COVID and myalgic encephalomyelitis/chronic fatigue syndrome (ME/CFS), particularly in symptoms such as post-exertional malaise, fatigue, and cognitive impairment [18]. Furthermore, SARS-CoV-2 has been hypothesized as a potential trigger for ME/CFS [19,20,21]. Based on this similarity, we considered the 180-day point to be a clinically meaningful threshold for distinguishing between recovery and progression to chronic illness in long COVID.

However, no longitudinal studies in Japan have comprehensively examined the clinical symptoms of long COVID and the background factors associated with symptom duration to date. Therefore, we conducted a retrospective cohort study to investigate the clinical characteristics and background factors related to the prolonged persistence of long COVID symptoms, with particular attention paid to the chronic phase beyond 180 days.

## 2. Materials and Methods

### 2.1. Study Design and Inclusion Criteria for Long COVID

This study was conducted as a retrospective cohort study. Outpatients who visited the COVID-19 aftercare outpatient clinic (CAC) at Okayama University Hospital and had their initial consultation between 1 February 2022, and 31 October 2024, were analyzed. Patients were included if they were aged 10 years or older, met the diagnostic criteria for long COVID at their initial visit, had a confirmed onset of COVID-19, and were infected with the Omicron variant. Long COVID was defined as symptoms persisting for 30 days or more after the onset of COVID-19 [21]. The Omicron variant was identified as COVID-19 infections that occurred in Okayama Prefecture on or after January 2022, on the basis of the websites of National Institute of Infectious Diseases, Japan. COVID-19: Changes in the SARS-CoV-2 Variants in Japan (Vol. 66, June 2022) at https://id-info.jihs.go.jp/niid/en/2019-ncov-e/10884-covid19-66-en.html (accessed on 3 July 2025) and Okayama Prefectural Government. Information on COVID-19 Variants in Okayama Prefecture at https://www.pref.okayama.jp/uploaded/attachment/366388.pdf (accessed on 3 July 2025). The patients were excluded if the physician at their initial visit determined that the primary complaint prompting the consultation was clearly attributable to a condition other than long COVID.

### 2.2. Data Collection

Data were obtained from electronic medical records at the CAC of Okayama University Hospital. The study included patients who received treatment at the CAC between 1 February 2022 and 30 April 2025, and whose initial visit to the CAC occurred between 1 February 2022 and 31 October 2024. The extracted data included age, gender, date of COVID-19 onset, date of first consultation, date of final medical visit, symptoms, history of alcohol consumption, smoking status, body mass index (BMI), vaccination history, severity of COVID-19 in the acute phase [22], laboratory test results, and questionnaire responses (the fatigue assessment scale [FAS], the Japanese version of the Euro QOL 5-dimension 5-level [EQ-5D-5L], and the self-rating depression scale [SDS]).

### 2.3. Patient Classification and Data Analysis

Medical records were analyzed during the data extraction period from 1 May 2025 to 22 May 2025. The duration of outpatient follow-up was calculated based on the initial and final consultation dates recorded in the medical records. Patients were classified into two groups according to their follow-up duration: those with a follow-up period of less than 180 days were categorized into the early recovery (ER) group, whereas those with a follow-up period of 180 days or more were classified into the persistent-symptom (PS) group.

30 April 2025 was designated as a provisional final consultation date for patients who were still receiving outpatient care at the time of analysis, and the follow-up duration was calculated accordingly. Comparative analyses between the ER and PS groups were conducted to evaluate the initial consultation age, gender, presenting symptoms, BMI, history of alcohol consumption, smoking status, vaccination history, severity of COVID-19 in the acute phase [22], laboratory test results, and questionnaire scores from the FAS [23,24], EQ-5D-5L [25], and SDS [26]. Furthermore, an exploratory analysis was performed to identify factors specifically associated with the PS group with the aim of determining characteristics linked to prolonged symptom persistence.

### 2.4. Laboratory Examination

Blood drawing was performed in the resting sitting position for each patient during a late morning outpatient visit. The blood samples were routinely analyzed using the auto-analyzer system in the central laboratory of our hospital. Laboratory data for hemoglobin (Hb), inflammatory markers (C-reactive protein: CRP and ferritin), liver functions (albumin: Alb, aspartate aminotransferase: AST, alanine aminotransferase: ALT), renal functions (creatinine: CRE), and low-density lipoprotein cholesterol (LDL-C) were obtained from medical records and analyzed in this study. Laboratory information on the following endocrine parameters was also obtained: adrenocorticotropin (ACTH), cortisol, free thyroxin (FT4), and thyrotropin (TSH). Plasma ACTH and serum cortisol were measured via an electro-chemiluminescence immunoassay (ECLIA) method using Elecsys ACTH and Elecsys Cortisol II kits (F. Hoffmann-La Roche AG, Basel Switzerland), respectively. Serum FT4 and TSH were determined with Elecsys FT4 III and Elecsys TSH kits (F. Hoffmann-La Roche AG), respectively. All laboratory tests used in this study were performed once for each item and conducted under strict quality control procedures in the central laboratory of our hospital which is accredited by the Japan Accreditation Board under ISO 15189 [27], the international standard for medical laboratories, which certifies competence and quality management systems. Therefore, the reliability and reproducibility of the test results are regularly ensured in accordance with international standards.

### 2.5. Assessment for Patients’ QOL and Mental Status

The patients’ quality of life (QOL) and mental health were assessed using the following standardized self-reported questionnaires, administered at the initial consultation. The Fatigue Assessment Scale (FAS) is a 10-item instrument designed to evaluate the severity of fatigue. It comprises five items on physical fatigue and five on mental fatigue, each rated on a 5-point Likert scale. Total scores range from 10 to 50, with higher scores indicating more severe fatigue [23,24]. The EuroQol 5-Dimension 5-Level (EQ-5D-5L) is a validated measure of health-related QOL that evaluates five dimensions: mobility, self-care, usual activities, pain/discomfort, and anxiety/depression. Each dimension is rated on a 5-level scale from “no problems” to “extreme problems,” and a health utility index score is calculated [25]. The Self-Rating Depression Scale (SDS) is a 20-item scale assessing depressive symptoms, with each item scored on a 4-point scale. The total score reflects the severity of depression, with higher scores indicating greater symptom burden [26].

### 2.6. Statistical Analysis

All statistical analyses were performed using Stata/SE 19 (StataCorp LLC, College Station, TX, USA) under an institutional license. Categorical variables are presented as numbers and percentages and were compared using Pearson’s χ^2^ test. Continuous variables are presented as medians with interquartile ranges (IQRs) and were analyzed using the Mann–Whitney U test due to non-normal distributions. *p* values of less than 0.05 were considered statistically significant.

### 2.7. Ethical Consideration

The research information regarding the present study was shown on our hospital website, and we offered patients the choice to opt out of participation in the study. Since all the patients’ data were anonymized, informed consent from each patient was not required. This study was conducted with the approval of the Okayama University Hospital Ethics Committee (No. 2105-030) and complied with the Declaration of Helsinki.

## 3. Results

First, the data for patients who visited our clinic during the study period were obtained from their medical records. During the study period, a total of 877 patients aged 10 years or older visited our clinic for their initial outpatient consultation. Among them, 103 patients were excluded for the following reasons: 33 patients had an unknown date of COVID-19 onset, 44 were infected with non-Omicron variants, 7 had no confirmed COVID-19 infection, 3 presented without any chief complaint at the initial visit, 11 were judged by the attending physician not to meet the clinical criteria for long COVID, and 5 were determined to have symptoms attributable to other underlying conditions based on the medical records. The remaining 774 patients with long COVID were analyzed in the present study (Table 1).

**Figure 1:**
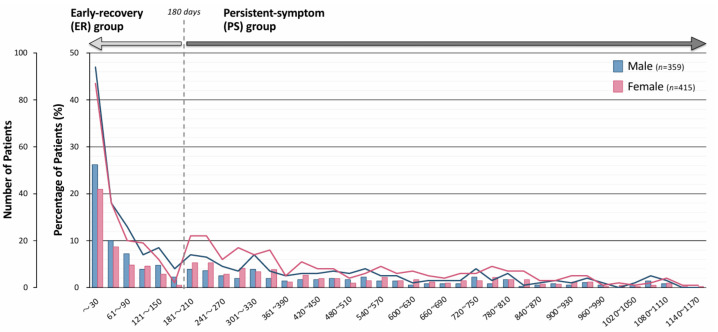
Number of patients according to outpatient follow-up duration. Line graph showing the number and bar graph showing the percentage of patients with long COVID according to the duration of outpatient follow-up (in days), stratified by gender. Follow-up durations are presented in 30-day intervals. The blue bars represent male patients, and the red bars represent female patients. Based on the distribution of follow-up durations for long COVID, patients were divided into two groups: those whose follow-up duration was less than 180 days were categorized into the early recovery (ER) group, and those whose duration was 180 days or more were categorized into the persistent-symptom (PS) group.

The distribution of durations for treating long COVID patients was analyzed. Figure 1 illustrates the distribution of outpatient follow-up durations among all 774 patients, stratified by gender. In both males and females, the highest proportion of patients discontinued follow-up within the first 30 days. Thereafter, the proportion of patients declined steadily across subsequent intervals up to 180 days. A modest increase was observed in the 181–360 days range, with a more noticeable peak in females than males. Following 360 days, proportion of patients in both groups remained consistently low. The longest follow-up duration was 1148 days in a female patient. The patients were classified into the early recovery (ER) group (*n* = 370, 47.8%) or the persistent-symptom (PS) group (*n* = 404, 52.2%), based on whether their follow-up duration exceeded 180 days (Figure 1).

As shown in Table 1, the median duration of outpatient treatment was 33 days [IQR: 1–72] in the ER group and 437 days [IQR: 280–707.5] in the PS group (** *p* < 0.01). There was no significant difference in median age between the ER and PS groups (40 [IQR: 24–53] vs. 42 [IQR: 28–52] years, respectively; *p* = 0.4055). However, a significant difference was observed in the gender distribution, with female patients comprising a higher proportion in the PS group than the ER group (59.4% vs. 47.3%, ** *p* < 0.01). No significant differences were found between the groups in terms of duration from infection to initial visit, smoking or alcohol habits, body mass index (BMI), or severity during the acute phase of COVID-19 (Table 1). Vaccination history was also assessed in relation to long COVID duration using four distinct grouping patterns: patients with no vaccination versus those with 1–7 doses, those with 0–1 dose versus 2–7 doses, those with 0–2 doses versus 3–7 doses, and finally those with 0–3 doses versus 4–7 doses. In all comparisons, there were no statistically significant differences between the ER and PS groups (Table 1).

**Figure 2:**
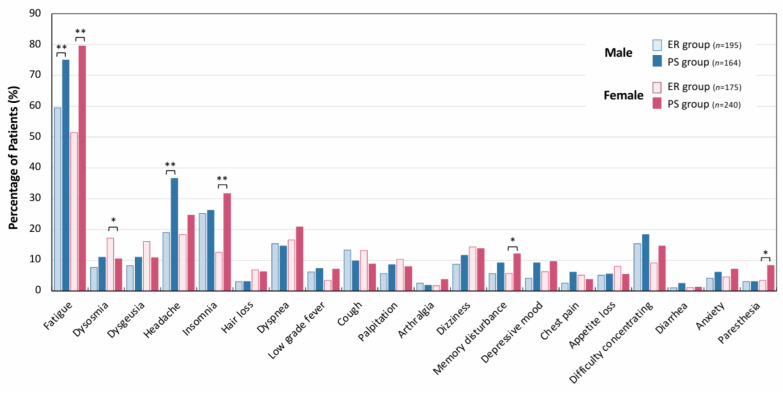
Symptomatic trends of long COVID patients based on recovery durations. The bar graphs show the percentage of patients who reported the representative 20 symptoms of long COVID. The blue bars represent male patients, and the red bars represent female patients. The light blue and red bars represent the early recovery (ER) group, while the dark blue and red bars indicate the persistent-symptom (PS) group. The data were analyzed by using the chi-squared test. * *p* < 0.05 and ** *p* < 0.01 were regarded as statistically significant.

Next, the symptomatic profiles and gender differences were examined in long COVID patients. As shown in Figure 2, the distribution of the 20 most common symptoms exhibited both group- and gender-dependent trends. Fatigue was the most common symptom across all groups, but the prevalence of fatigue symptoms was significantly higher in the PS group than the ER group (** *p* < 0.01). Among female patients, the PS group had markedly higher prevalence rates of insomnia, memory disturbance, and paresthesia than the ER group (** *p* < 0.01 for insomnia, * *p* < 0.05 for memory disturbance and paresthesia). Among male patients, the prevalence of headache was significantly higher in the PS group than in the ER group (** *p* < 0.01).

**Figure 3:**
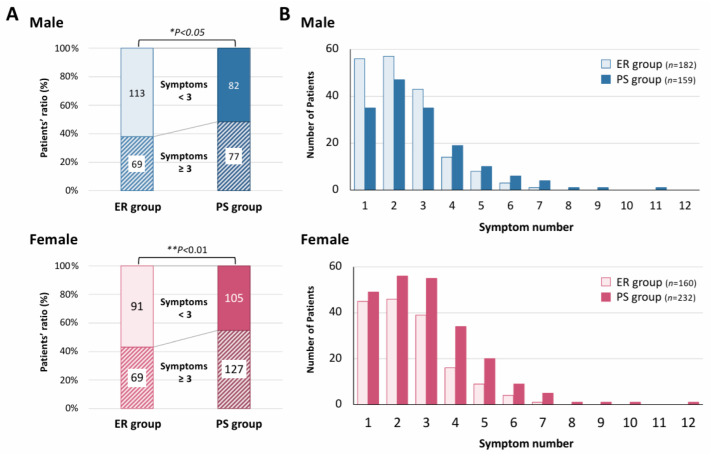
Relationship between number of long COVID symptoms and symptom durations. (**A**) The panels show the ratios of patients who reported less than 3 and those reporting 3 or more symptoms out of the 20 representative symptoms shown in Figure 2 between the ER and PS groups. The upper panel shows data for male patients, and the lower panel shows data for female patients. Statistical analysis was performed using the Mann–Whitney U test; * *p* < 0.05 and ** *p* < 0.01 were considered statistically significant. (**B**) The graphs show the number of long COVID symptoms reported by the patients ranging from 1 to 12 out of the 20 representative symptoms shown in Figure 2. In the figure, data for male and female patients are presented in the upper and lower panels, respectively. The graphs also demonstrate the gender-dependent distributions of patients in the ER and PS groups.

Figure 3 illustrates the association between the number of representative symptoms at the initial visit and the duration of outpatient follow-up, focusing on the 20 most common symptoms shown in Figure 2. Among the 195 male patients in the ER group, 182 presented with at least one of these 20 symptoms. Similarly, among the 175 female patients in the ER group, 160 reported these symptoms. In the PS group, 159 of the 164 male patients and 232 of the 240 female patients exhibited one or more of the 20 representative symptoms. Among male patients, 37.9% (69 of 182 patients) of those in the ER group presented with three or more symptoms out of the 20 representative symptoms shown in Figure 2 at the initial visit, whereas this proportion was significantly higher in the PS group at 48.4% (77 of 159 patients; * *p* < 0.05). This trend was more pronounced among female patients, with 43.1% (69 of 160 patients) in the ER group exhibiting three or more symptoms, compared to 54.7% in the PS group (127 of 232 patients; ** *p* < 0.01; Figure 3A). Furthermore, Figure 3B details the distribution of symptom counts out of the 20 representative symptoms shown in Figure 2. In the ER group, both male and female patients predominantly presented with one to two symptoms whereas, in the PS group, two to three symptoms were more common. Notably, among female patients in the PS group, more than 20 individuals exhibited 4–5 symptoms of long COVID. The number of long COVID patients with a single symptom at their first visits was also clarified. In the PS group, a majority of both male and female patients complained of fatigue (Supplement Figure S1). While fatigue was the most common persistent symptom in both genders, dysosmia and dyspnea were the second most common among male and female patients of the PS group, respectively (Supplement Figure S1). On the other hand, in the ER group, 12 and 13 different types of symptoms in male and female patients, respectively, were detected in our study.

**Figure 4:**
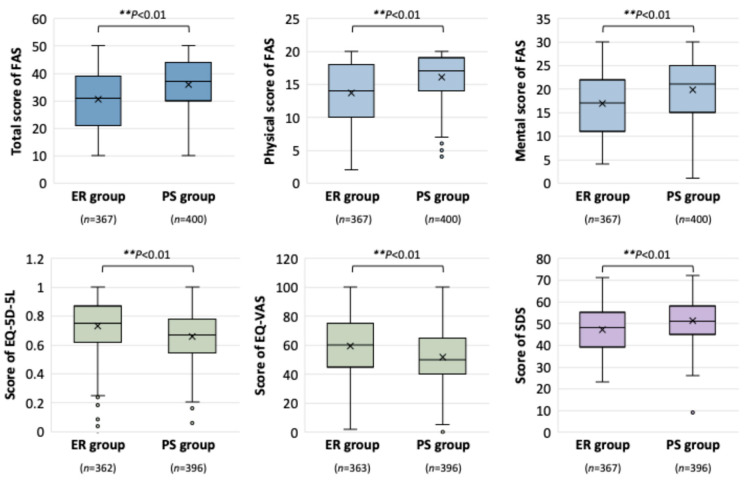
Comparison of baseline scores of self-assessed scales for fatigue, quality of life, and depression and symptom durations in long COVID patients. Box plots depict the median, interquartile range (IQR), and values within 1.5 × IQR; the mean is marked with an “×”. Statistical analysis was performed using the chi-squared test; ** *p* < 0.01 were considered statistically significant. Abbreviations: FAS, fatigue assessment scale; EQ-5D-5L, Euro QOL 5-dimension 5-level; EQ-VAS, Euro QOL visual analog scale; SDS, self-rating depression scale.

Furthermore, the effects of therapeutic durations on the self-assessment of fatigue, QOL, and depression were examined. Long COVID patients in the PS group demonstrated significantly worse scores in standardized self-reported scales assessing fatigue, quality of life, and depression (Figure 4). Valid responses were obtained from 367 ER and 400 PS patients for the FAS; 362 ER and 396 PS patients for the EQ-5D-5L; 363 ER and 396 PS patients for the EQ-VAS; and 367 ER and 396 PS patients for the SDS. The total FAS score was significantly higher in the PS group (median: 37.0 [IQR 30.0–44.0]) than the ER group (31.0 [IQR 21.0–39.0]; ** *p* < 0.01). Individually, the physical FAS scores were higher in the PS group (median: 17.0 [IQR 14.0–19.0]) than in the ER group (median: 14.0 [IQR 10.0–18.0]; ** *p* < 0.01). Similarly, mental fatigue scores were significantly higher in the PS group (median: 21.0 [IQR 15.0–25.0]) than in the ER group (median: 17.0: [IQR 11.0–22.0]; ** *p* < 0.01). Quality of life, as measured with the EQ-5D-5L, was lower in the PS group (0.67 [IQR 0.54–0.78]) than in the ER group (0.75 [IQR 0.62–0.87]; ** *p* < 0.01), and the EQ-VAS scores showed a similar trend (PS: 50.0 [IQR 40.0–65.0] vs. ER: 60.0 [IQR 45.0–75.0]; ** *p* < 0.01). Further, the scores for depression status according to the SDS were significantly elevated in the PS group (51.0 [IQR 45.0–58.0]) versus the ER group (48.0 [IQR 39.0–55.0]; ** *p* < 0.01).

Regarding the laboratory findings in the patients with long COVID, Table 2 summarizes the baseline laboratory findings for both male and female patients. Most values remained within normal reference ranges and showed no significant differences between the ER and PS groups. No significant differences were observed between the two groups in terms of hemoglobin levels, liver functions (Alb, AST, and ALT), inflammatory responses (CRP and ferritin, although ferritin has been reported to increase with the prolongation of long COVID) [28], thyroid hormone levels (TSH and FT4), or adrenocortical hormone levels (ACTH and cortisol) in either men or women. However, LDL-C levels were significantly higher in male PS patients (median: 124 mg/dL [IQR 100–151]) than male ER patients (111 mg/dL [IQR 92–136]; ** *p* < 0.01), and were also slightly elevated in female PS patients (120 mg/dL [IQR 97–143]) compared to ER (111 mg/dL [IQR 92–135]; * *p* < 0.05) (Table 2). Additionally, female patients in the PS group showed a statistically lower creatinine level (0.60 mg/dL [IQR 0.54–0.67]) than those in the ER group (0.62 mg/dL [IQR 0.56–0.72]; ** *p* < 0.01), although remaining within physiological limits.

## 4. Discussion

In the present study, we revealed that more than half of the patients attending a long COVID outpatient clinic at a university hospital in Japan continued follow-up for over 180 days (6 months). The patients in the group with follow-up durations exceeding 180 days were more likely to be female, had a greater number of symptoms at the initial visit, and reported higher levels of fatigue, lower QOL, and poorer mental health status at baseline. In terms of specific symptoms, women with a follow-up period longer than 180 days were significantly more likely to report fatigue, insomnia, memory disturbance, and paresthesia, while men in the same group reported a higher frequency of headaches. Regarding the laboratory values, no obvious differences in blood test data were observed even in cases with longer follow-up periods than 180 days. However, both male and female patients with a long follow-up period had higher LDL-C levels.

Several prior studies based on internet surveys have reported that symptoms of long COVID can persist for 6 months to 1 year [13,29,30,31], with some cases extending up to 2 years [32]. The present study, conducted from the perspective of clinicians in a medical setting, supports these findings. Our results align with previous research indicating that fatigue, brain fog, and headache tend to be more persistent [13,29,30,31,32]. In this study, by extending the observation period to six months, we further highlight the need for prolonged clinical management in patients with long COVID. In addition, even a single symptom of long COVID can persist for an extended period. While fatigue was the most common persistent symptom in both genders, dysosmia and dyspnea were the second most common among male and female patients, respectively. Whether these differences reflect sex-based variations in the affected organ systems or differences in the perception of and sensitivity to symptoms remains to be elucidated.

Notably, female gender has been identified as a risk factor for long COVID in several previous studies [13,31,32,33]. The present study also revealed that women accounted for the majority (59.4%) of the patients with prolonged long COVID, and even those with few symptoms may have developed prolonged long COVID. In this regard, we have also previously reported that long COVID is more prevalent among women in their 40 s and that menstrual irregularities accompanied by depressive symptoms increased following infections during the Omicron-dominant phases [34]. The increased risk of long COVID among women is thought to be influenced by both biological and social factors [35]. One proposed biological mechanism is that a decline in estrogen may contribute to immune dysregulation. In addition, it has been suggested that the COVID-19 pandemic has exacerbated gender disparities [36,37]. In Japan, there have also been indications of worsening mental health among women and changes in lifestyle patterns [7].

The long COVID patients with persistent symptoms also exhibited elevated FAS scores, including mental fatigue components, as well as higher SDS scores and lower EQ-5D-5L index values in the present study. These findings suggest that poor mental health may be one of the contributing factors to the prolonged course of long COVID. Another notable finding was derived from blood biochemical data: LDL-C levels tended to be elevated in the symptom-persistent group. Basic research has suggested that dyslipidemia may impair immune responses and promote chronic inflammation [38,39,40], supporting the potential link between lipid metabolism abnormalities and long COVID. Further studies are warranted to explore this association in greater depth. On the other hand, a large-scale cohort study has also demonstrated that COVID-19 survivors had a significantly higher risk of developing dyslipidemia in one year, including elevated LDL-C levels, compared with matched uninfected controls [41]. These findings suggest that post-COVID dyslipidemia is not uncommon and may reflect ongoing metabolic and inflammatory dysregulation [41]. Further studies including the potential impacts of lipid abnormalities on the persistence of long COVID symptoms are needed to explore this association.

Another intriguing finding of this study is the identification of approximately 180 days as a potential watershed point distinguishing between self-limited and prolonged courses of long COVID among Japanese patients. The group with symptoms persisting beyond 180 days also exhibited a clear gender difference, with a predominance of female patients. Moreover, the continuation of long COVID symptoms and the persistence of fatigue for 180 days (6 months) is key to meeting several diagnostic criteria for myalgic encephalomyelitis/chronic fatigue syndrome (ME/CFS) [21]. In fact, in the clinical courses of long COVID patients, we have reported that 8.4% of patients have progressed to a condition that meets the diagnostic criteria for ME/CFS [21]. Women particularly at risk for long-term symptoms may require careful and comprehensive management from the early stages of care, especially for symptoms such as fatigue, insomnia, memory impairment, and paresthesia. In men, the presence of comorbid headache may also necessitate early and proactive intervention as it has been associated with prolonged symptoms and reduced QOL, as reported in a previous study [42]. These individuals often presented with multiple complaints at the initial visit and showed impaired self-reported assessments of fatigue, QOL, and mental health. Such characteristics suggest that the persistence of symptoms may reflect the manifestation or prolongation of pre-existing subclinical conditions, potentially influenced by some gender-related factors.

A major strength of this study lies in its methodology. While many previous studies have relied on internet-based surveys and patient self-reports to assess symptoms, our study utilized data obtained from face-to-face clinical encounters, allowing for objective symptom evaluation by physicians. Furthermore, blood tests were used to exclude other overt medical conditions, enhancing the validity of the findings. The absence of significant differences in hypothalamic–pituitary–adrenal (HPA) axis function, thyroid function, complete blood counts, liver function, and electrolyte levels between the groups likely reflects the appropriate clinical exclusion of other diagnoses. This suggests that cases mimicking long COVID were effectively ruled out by attending physicians. Another notable advantage is that, whereas many earlier studies focused on infections caused by the ancestral strain through the Delta variant, this study exclusively examined cases during the Omicron-dominant period, providing updated and relevant insights into the current phase of the pandemic. Regarding vaccination conditions, the number of vaccinations between the two groups was carefully compared, and it was confirmed that there was no clear correlation in the prolongation of symptoms.

Nevertheless, this study has several limitations. Unlike many previous studies that included community-based or mild cases, our study population consisted of patients referred to a university hospital specialty clinic, which may have introduced a selection bias toward more severe or treatment-resistant cases. Additionally, we did not assess potential confounding factors such as pre-existing conditions or ongoing comorbidities. Long COVID has a wide variety of symptoms for each case, making it difficult to use disease- or pathogen-specific drugs and requiring careful symptomatic treatment for each case. In this study, treatment interventions were tailored to each individual case based on symptoms. Although clinical decisions were shared within the care team through regular conferences, a major limitation of the study is that treatment strategies were not standardized. Furthermore, due to the nature of university hospitals in Japan, once a treatment plan is formulated, ongoing care is often handed over to primary care providers. As a result, it was difficult to track symptomatic changes after patients discontinued follow-up at our institution, which may have limited our ability to accurately evaluate the true duration of long COVID symptoms.

## 5. Conclusions

Based on the present study, long COVID patients with more than three symptoms were predominant in the prolonged cases (i.e., those longer than 180 days) in both genders. The patients with persistent symptoms had significantly higher scores for physical and mental fatigue as well as for depressive symptoms. Thus, long-lasting long COVID could be linked to the number of symptoms, albeit in a gender-dependent manner.

## Figures and Tables

**Table 1:** Background of long COVID patients during the Omicron phase.

Duration of Outpatient Treatment		Early Recovery (ER) Group (*n* = 370)		Persistent-Symptom (PS) Group (*n* = 404)		*p*-Value
Duration (median days, [IQR])		33	[1–72]	437	[280–707.5]	<0.01 ^(a)^**
Number of patients (%)		370	(47.8)	404	(52.2)	
Age years, median [IQR]		40	[24–53]	42	[28–52]	0.4055 ^(a)^
Gender, *n* (%)						
	Male	195	(52.7)	164	(40.6)	<0.01 ^(b)^**
	Female	175	(47.3)	240	(59.4)	
Interval (infection to the visit), median [IQR]		103	[65–167]	99	[62–189]	0.8824 ^(a)^
Smoking habits, *n* (%)		89	(24.3)	107	(26.8)	0.626 ^(b)^
Alcohol habits, *n* (%)		100	(27.4)	90	(22.6)	0.127 ^(b)^
BMI, median [IQR]		22.5	[20.0–25.8]	21.7	[19.4–25.3]	0.1215 ^(a)^
Severity of acute condition, *n* (%)						
	Mild (%)	360	(97.3)	393	(97.3)	0.997 ^(b)^
	Moderate (%)	9	(2.4)	10	(2.5)	
	Severe (%)	1	(0.2)	1	(0.25)	
Vaccinations, number (%)						
	0 (%)	82	(22.6)	95	(23.7)	0.719 ^(b)^
	≥1~7 (%)	281	(77.4)	306	(76.3)	
	0~1 (%)	84	(23.1)	100	(24.9)	0.562 ^(b)^
	≥2~7 (%)	279	(76.9)	301	(75.1)	
	0~2 (%)	192	(52.9)	200	(49.9)	0.405 ^(b)^
	≥3~7 (%)	171	(47.1)	201	(50.1)	
	0~3 (%)	287	(79.1)	312	(77.8)	0.673 ^(b)^
	≥4~7 (%)	76	(20.9)	89	(22.2)	

Patients were categorized into the early recovery (ER) group or the persistent-symptom (PS) group according to whether their long COVID recovery time was shorter or longer 180 days, respectively. Medians [IQR: interquartile range] and percentages (%) are shown. *p*-values reflect differences between the ER and PS groups. The data were analyzed using (a) the Mann–Whitney U test or (b) the chi-squared test. ** *p* < 0.01 were regarded as statistically significant.

**Table 2:** Laboratory data of long COVID patients in the two groups.

Male Patients		(ER) Group (*n* = 195)		(PS) Group *(n* = 164)		*p*-Value
	Reference Range	Median [IQR]	*n*	Median [IQR]	*n*	
Hb (g/dL)	13.7–16.8	15.4 [14.7–16.0]	160	15.4 [14.4–16.1]	158	0.4276
Alb (g/dL)	4.1–5.1	4.6 [4.3–4.8]	156	4.6 [4.3–4.8]	157	0.9530
AST (U/L)	13–30	19 [16–25]	159	21 [17–27]	158	0.1730
ALT (U/L)	10–42	21 [14–31]	159	24 [16–40]	158	0.0599
CRE (mg/dL)	0.65–1.07	0.84 [0.76–0.92]	159	0.82 [0.75–0.91]	158	0.4294
LDL-C (mg/dL)	65–163	111 [92–136]	148	124 [100–151]	151	<0.01 **
Ferritin (ng/mL)	39.9–465	200 [117–308]	157	210 [129–338]	158	0.6949
CRP (mg/dL)	<0.15	0.05 [0.02–0.11]	161	0.06 [0.03–0.13]	158	0.0776
TSH (mIU/L)	0.61–4.23	1.52 [1.00–2.09]	158	1.48 [0.97–2.29]	152	0.8791
FT4 (ng/dL)	0.97–1.69	1.35 [1.22–1.48]	158	1.3 [1.16–1.46]	152	0.1252
ACTH (pg/mL)	7.2–63.3	25.8 [19.1–38.9]	155	23.7 [17.9–33.6]	155	0.0952
Cortisol (μg/dL)	7.1–19.6	7.4 [5.2–11.0]	155	7.2 [5.1–10.1]	155	0.5111
Female Patients		ER Group (*n* = 175)		PS Group (*n* = 240)		*p*-Value
	Reference range	Median [IQR]	*n*	Median [IQR]	*n*	
Hb (g/dL)	11.6–14.8	13.55 [12.9–14.3]	142	13.50 [12.8–14.1]	221	0.2225
Alb (g/dL)	4.1–5.1	4.4 [4.2–4.6]	138	4.4 [4.2–4.6]	221	0.7997
AST (U/L)	13–30	18 [15–23]	140	18 [15–21]	220	0.6029
ALT (U/L)	7–23	16 [11–23]	140	14 [11–21]	220	0.1753
CRE (mg/dL)	0.65–1.07	0.62 [0.56–0.72]	139	0.60 [0.54–0.67]	220	<0.01 **
LDL-C (mg/dL)	65–163	111 [92–135]	131	120 [97–143]	212	<0.05 *
Ferritin (ng/mL)	6.2–138	71.4 [36.9–150]	137	67.9 [29.0–125]	220	0.1332
CRP (mg/dL)	<0.15	0.05 [0.02–0.16]	141	0.05 [0.02–0.10]	221	0.6109
TSH (mIU/L)	0.61–4.23	1.42 [0.89–2.24]	136	1.52 [1.04–2.07]	218	0.8126
FT4 (ng/dL)	0.97–1.69	1.22 [1.105–1.36]	136	1.28 [1.13–1.38]	218	0.4130
ACTH (pg/mL)	7.2–63.3	18.2 [11.5–26.0]	138	16.4 [12.0–22.9]	220	0.1640
Cortisol (μg/dL)	7.1–19.6	8.1 [5.7–10.7]	138	7.3 [5.4–10.1]	220	0.2564

ER: early recovery; PS: persistent-symptom. The reference range represents the range that includes 95% of healthy adult Japanese individuals. Medians [IQR: interquartile range] and percentages (%) are shown. *p*-values reflect differences between the ER and PS groups. The data were analyzed using the Mann–Whitney U test. * *p* < 0.05 and ** *p* < 0.01 were regarded as statistically significant.

## Data Availability

Detailed data will be available upon request to the corresponding author.

## Supplementary Materials

The following supporting information can be downloaded at: https://www.mdpi.com/article/10.3390/jcm14144918/s1, Supplement Figure S1. Characteristics of long COVID patients with one symptom and their symptom durations. Each bar graph represents the ratio of patients exhibiting the specific symptom as only a single symptom at the initial visit. The graphs are divided into the early recovery (ER) group and the persistent-symptom (PS) group, with male patients in the upper portion and female patients in the lower portion. The symptoms correspond to the 20 types of long COVID symptoms shown in Figure 2.

## Abbreviations

body mass index (BMI); coronavirus disease 2019 (COVID-19); COVID-19 aftercare clinic (CAC); Euro QOL 5-dimension 5-level (EQ-5D-5L); fatigue assessment scale (FAS); early recovery group (ER group); low-density lipoprotein cholesterol (LDL-C); myalgic encephalomyelitis/chronic fatigue syndrome (ME/CFS); persistent-symptom group (PS group); post-COVID-19 condition (PCC); quality of life (QOL); self-rating depression scale (SDS); visual analog scale (VAS).
