# Supplementary material for: Symptomatic Trends and Time to Recovery for Long COVID Patients Infected During the Omicron Phase
Source: J Clin Med. 2025 Jul 11;14(14):4918. doi: 10.3390/jcm14144918 (PMC12295900; doi:10.3390/jcm14144918)
Supplement: Supplementary file 1 [file jcm-14-04918-s001.zip › jcm-3729658-supplementary.pdf]

Suppl. Fig. 1

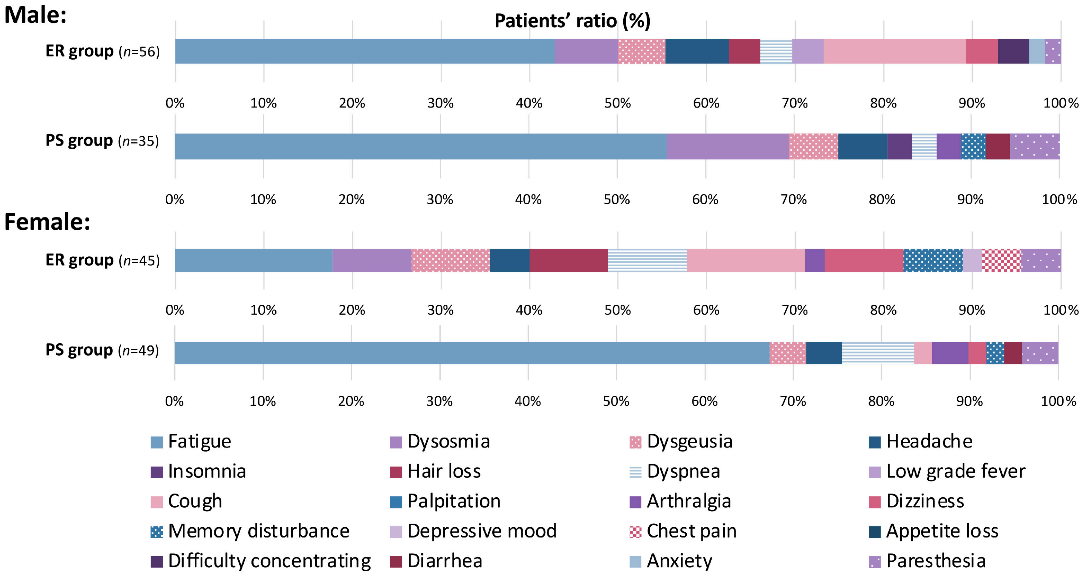

Supplement Figure S1. Characteristics of long COVID patients with one symptom and their symptom durations. Each bar graph represents the ratio of patients exhibiting the specific symptom as only a single symptom at the initial visit. The graphs are divided into the early recovery (ER) group and the persistent-symptom (PS) group, with male patients in the upper portion and female patients in the lower portion. The symptoms correspond to the 20 types of long COVID symptoms shown in Figure 2.
